# High copy number variations, particular transcription factors, and low immunity contribute to the stemness of prostate cancer cells

**DOI:** 10.1186/s12967-021-02870-x

**Published:** 2021-05-13

**Authors:** Zao Dai, Ping Liu

**Affiliations:** College of Life Sciences, Nanjing Normal University, Nanjing, Jiangsu China

**Keywords:** Stemness of prostate cancer, WGCNA, ATAC-seq, CNV, Immune infiltration

## Abstract

**Background:**

Tumor metastasis is the main cause of death of cancer patients, and cancer stem cells (CSCs) is the basis of tumor metastasis. However, systematic analysis of the stemness of prostate cancer cells is still not abundant. In this study, we explore the effective factors related to the stemness of prostate cancer cells by comprehensively mining the multi-omics data from TCGA database.

**Methods:**

Based on the prostate cancer transcriptome data in TCGA, gene expression modules that strongly relate to the stemness of prostate cancer cells are obtained with WGCNA and stemness scores. Copy number variation of stemness genes of prostate cancer is calculated and the difference of transcription factors between prostate cancer and normal tissues is evaluated by using CNV (copy number variation) data and ATAC-seq data. The protein interaction network of stemness genes in prostate cancer is constructed using the STRING database. Meanwhile, the correlation between stemness genes of prostate cancer and immune cells is analyzed.

**Results:**

Prostate cancer with higher Gleason grade possesses higher cell stemness. The gene set highly related to prostate cancer stemness has higher CNV in prostate cancer samples than that in normal samples. Although the transcription factors of stemness genes have similar expressions, they have different contributions between normal and prostate cancer tissues; and particular transcription factors enhance the stemness of prostate cancer, such as PUM1, CLOCK, SP1, TCF12, and so on. In addition, the lower tumor immune microenvironment is conducive to the stemness of prostate cancer. CD8 + T cells and M1 macrophages may play more important role in the stemness of prostate cancer than other immune cells in the tumor microenvironment. Finally, EZH2 is found to play a central role in stemness genes and is negatively correlated with resting mast cells and positively correlated with activated memory CD4 + T cells.

**Conclusions:**

Based on the systematic and combined analysis of multi-omics data, we find that high copy number variation, specific transcription factors, and low immune microenvironment jointly contribute to the stemness of prostate cancer cells. These findings may provide us new clues and directions for the future research on stemness of prostate cancer.

**Supplementary Information:**

The online version contains supplementary material available at 10.1186/s12967-021-02870-x.

## Background

Cancer stem cells (CSCs) are a few stemness-like cells with the ability of self-renewal and differentiation in cancers [1]. They play an important role in the occurrence and development of cancers, especially closely related to cancer metastasis [2–4]. In prostate cancer, the stemness of cancer cells (including prostate cancer stem cells, PCSC) is closely related to the metastasis of prostate cancer [5]. In the metastasis process of prostate cancer, PCSCs initiates EMT (epithelial-mesenchymal transition) to form fibroblast-like cells and then enter the blood. With the circulation system, prostate cancer cells migrate to other tissues (such as bone tissue and lymph tissue) and grow into tumor tissues, which leads to tumor metastasis (cancer cell spreading).

As we known, there are many factors which relate to the stemness of CSC cells, including both intracellular factors (such as stemness-related genes) and microenvironment of cancer tissues (such as immune cells in the tumor microenvironment) [3, 6, 7]. In prostate cancer (PCa), it has been reported that the immune cells (especially CD8 + T cells and macrophages) in the microenvironment of PCa are closely related to the metastasis of PCa cells [8, 9]. The number of immune cells around the early PCa tissue will be decreased with the growth of the cancer tissue, which results in the decrease of the immunity in the microenvironment of PCa [10]. With the development of PCa to the later stage (Gleason score to 6–10), some immune cells (such as related T cells) in the microenvironment can reverse to promote or enhance the growth of PCa and help cancer cell metastasis [11].

Although increasing evidences have shown the relationship between stemness and metastasis in PCa cells, few studies on the stemness regulation of PCa cells are reported [12, 13]. Lots of factors affecting the stemness of PCa cells remain unclear and need to be investigated.

The bioinformatics method based on the TCGA database has been increasingly used to analyze the molecular basis of prostate cancer development and clinical patient prognosis [14–16]. Using appropriate analysis software and methods to explore a variety of large-size data of clinical specimen from the TCGA database (including transcriptome sequencing data, gene sequencing data, ATAC-seq data, etc.), the molecular basis of prostate tumorigenesis, development of PCa, and the prognosis of patients may be figured out [17, 18]. Therefore, the bioinformatics analysis of TCGA data can provide clues and directions for both the basic experimental research and the clinical cancer treatment in PCa.

In this study, we firstly obtained the most important stemness-related modules and genes in prostate cancer cells from transcriptome data and OCLR scores (a method for scoring the stemness of tumors) [2]. Further analysis showed that stemness-related genes had higher CNV in prostate cancer than that in prostate normal samples. Transcription factors of stemness genes enhanced the stemness of prostate cancer cells. The stemness of prostate cancer cells was negatively correlated with the immune response; and low immune scores were beneficial to the prostate cancer stemness. EZH2 was found to play a central role in these stemness genes. All our multi-omics analyzing results might provide some theoretical clues for us to experimentally investigate the factors affecting the PCa cell stemness and its relationships between PCSC and PCa metastasis.

## Methods

### Analysis of clinical data

We obtained the clinical information of prostate cancer from TCGA database and then divided prostate cancer samples into five grades according to the Gleason score and named Gleason grade 1 (Gleason score 6), Gleason grade 2 (Gleason score (3 + 4)), Gleason grade 3 (Gleason score (4 + 3)), Gleason grade 4 (Gleason score 8), Gleason grade 5 (Gleason score 9 or 10), respectively.

### Analysis of transcriptome (RNA-seq) data

Based on OCLR stemness scores and Gleason classification of TCGA prostate cancer clinical data, correlation analysis between PCa cell stemness and Gleason grade was carried out. By combing the stemness score, WGCNA [19] analysis was performed on the transcriptome data of prostate cancer in TCGA. Differential expression analysis of genes in the WGCNA results that mostly related to PCa cell stemness was performed and presented in a heatmap. Further, the normalized expression of stemness genes was obtained by using between-array normalization of the limma [20] package to analyze the transcriptome data of 33 samples (GSE104786) from GEO database and then presented in a heatmap. Also, the transcriptome data from SRA database (Normal samples SRR7651698, SRR7651699, SRR7651700), PCa cell lines (SRR7651715, SRR7651716, SRR7651717, SRR7651718), and other small cell prostate cancer (SRR7651719, SRR7651720) were aligned and quantified by using HISAT2 [20] and HTSeq [21], respectively. Finally, the normalized expression data was obtained by using TPM (Transcript per million) normalization method.

### Analysis of gene CNV data

The genes most related to stemness in WGCNA results were screened, and their locations in genome were obtained by local Perl script method. Combined with the CNV data of TCGA, the local Perl script was used to screen the segments containing the locations of important stemness genes. The prostate cancer samples were classified by Gleason grade and then the CNVs of essential stemness genes in each type of prostate cancer sample were calculated by using GISTIC2.0 [22].

### Analysis of ATAC-seq data

From TCGA and SRA database, we got the ATAC-seq data, including normal prostate samples (SRR7651660, SRR7651661, SRR7651662), prostate cancer samples (SRR7651675, SRR7651676), and TCGA ATAC-seq alignment results of prostate cancer samples. Then 2 kb data of upstream and downstream of the important stemness genes were obtained by using Bowtie2 software [23]. According to the results, the TSS signal intensity of stemness genes was drown by using deeptools [24]. After analyzing the above alignment data using MACS [25] and HOMER [26], information about transcription regulators of the stemness genes was obtained; and the importance of each transcriptional factor was further obtained from TCGA transcriptome data by PCA analysis with R language. For example, the upstream transcriptional factor of EZH2 was drawn using Sushi [27] package in R language.

### Analysis of tumor microenvironment and immune infiltration

The tumor microenvironment score and tumor immune infiltration score were calculated by using ESTIMATE [28] and CIBERSORT [29], respectively. Then, the correlation between stemness score and the immune score of PCa cells was calculated with R by combining with Gleason grade of prostate cancer. Based on CIBERSORT analysis results, the distribution map of immune cell components of different Gleason grades in prostate cancer was drawn with R language. The correlation network among immune cells was also drawn with R language according to the correlation and significance of different immune cells.

### Protein interaction network and the correlation between stemness genes and immune infiltration

After getting and analyzing PPI (Protein–Protein Interaction) data from the STRING (functional protein association networks) database, the PPI data of essential stemness genes were obtained. The interaction network of proteins of essential stemness genes was drawn with Cytoscape [30]. GO and KEGG enrichment analysis of important stemness genes were also carried out by using ClusterProfiler [31]. Finally, by using R language to jointly analyze and calculate the transcription data of essential stemness genes and the consequences of immune infiltration, the correlation between the critical stemness genes and immune cells was obtained.

## Results

### Stemness-related key genes are highly expressed in high Gleason grade prostate cancer samples

**Fig. 1:**
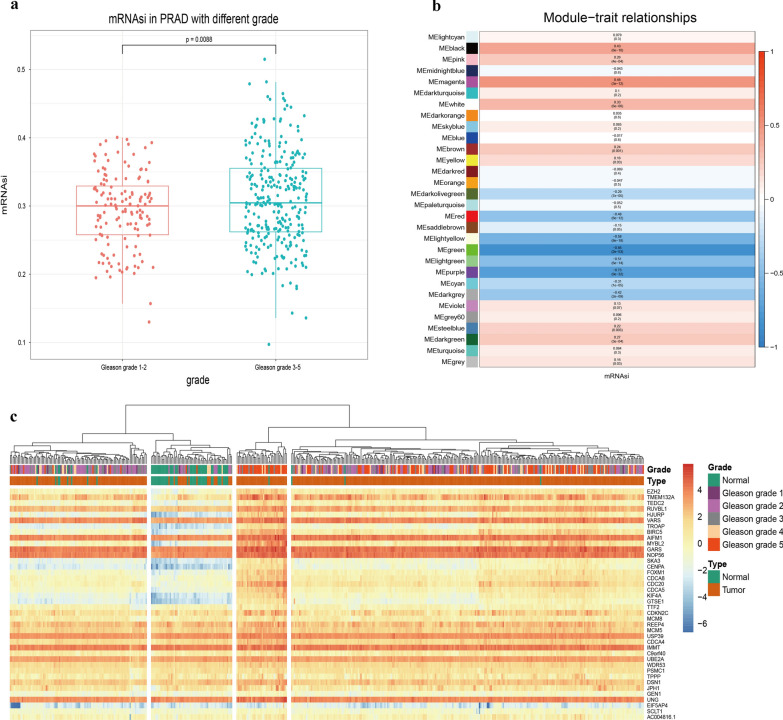
The relationship between stemness score and module and Gleason grade. **a** The relationship between prostate cancer stemness score and Gleason grade. **b** The gene module related to stemness was analyzed by the WGCNA method. Red represented a positive correlation between gene module and stemness, and blue represented a negative correlation between gene module and stemness. **c** The heatmap of genes in MEmagenta module. Red represents high gene expression, and blue represents low gene expression

Based on the joint analysis of the OCLR's results and transcriptome data of PCa clinical specimen in TCGA, it’s found that the stemness of PCa cells of Gleason grade 3–5 was significantly higher than that of Gleason grade 1–2 (Fig. 1a), Besides, by using WGCNA (Weighted Gene Co-expression Network Analysis) to analyze the PCa transcriptome data for cancer cell stemness-related gene transcripts, we got 30 transcript modules related to PCa cell stemness (Fig. 1b), and samples were clustered into different modules (Additional file 1: Fig. S1a.). About 88.2% of the 2158 known cell stemness-related genes [32] were found in the WGCNA analysis results (Additional file 2: Table S1; Additional file 1: Fig. S1c). Among these modules, we found that the MEmagenta module had the most positive correlation with the stemness of prostate cancer (Fig. 1b: Additional file 1: Fig. S1b), suggesting that the genes in the MEmagenta module (Additional file 3: Table S2) might be important and play a vital role in the stemness of prostate cancer.

Further analysis showed that the expression of genes in the MEmagenta module in PCa cancer specimen was generally higher than that in the normal prostate specimen; and the expression of these stemness genes in PCa increased with Gleason grade, and the highest expression was found at Gleason grade 5 (Fig. 1c). In addition, we analyzed the data from 33 samples of GEO database and found that expressions of the most stemness genes in small cell prostate cancer (SCPC) were higher than those in prostate adenocarcinoma (PRAD) (Additional file 1: Fig. S1d). Meanwhile, analysis of the transcriptome data of different prostate (normal or PRAD) cell lines and SCPC cell lines was also carried out, and it's found that the expression of stemness gene in PCa cells (PRAD cell lines) was higher than that in normal cells, as well as the expression of stemness genes in SCPC cell lines was slightly higher than in PRAD cell lines (Additional file 1: Fig. S1e).

In the genes of the MEmagenta module (Additional file 3: Table S2), we found that the known genes closely related to cancer stemness in non-prostate cancer, such as BRCA1 [33], EZH2 [33], FOXM1 [34], CDC20 [35], and CDCA8 [36], were all clustered in this module, indicating these genes were also closely related to the stemness of prostate cancer cells. From the data of Additional file 3, it’s confirmed that EZH2 was the most significant gene in the correlation with the stemness of PCa cells.

### The CNV of stemness-related genes was elevated in prostate cancer

**Fig. 2:**
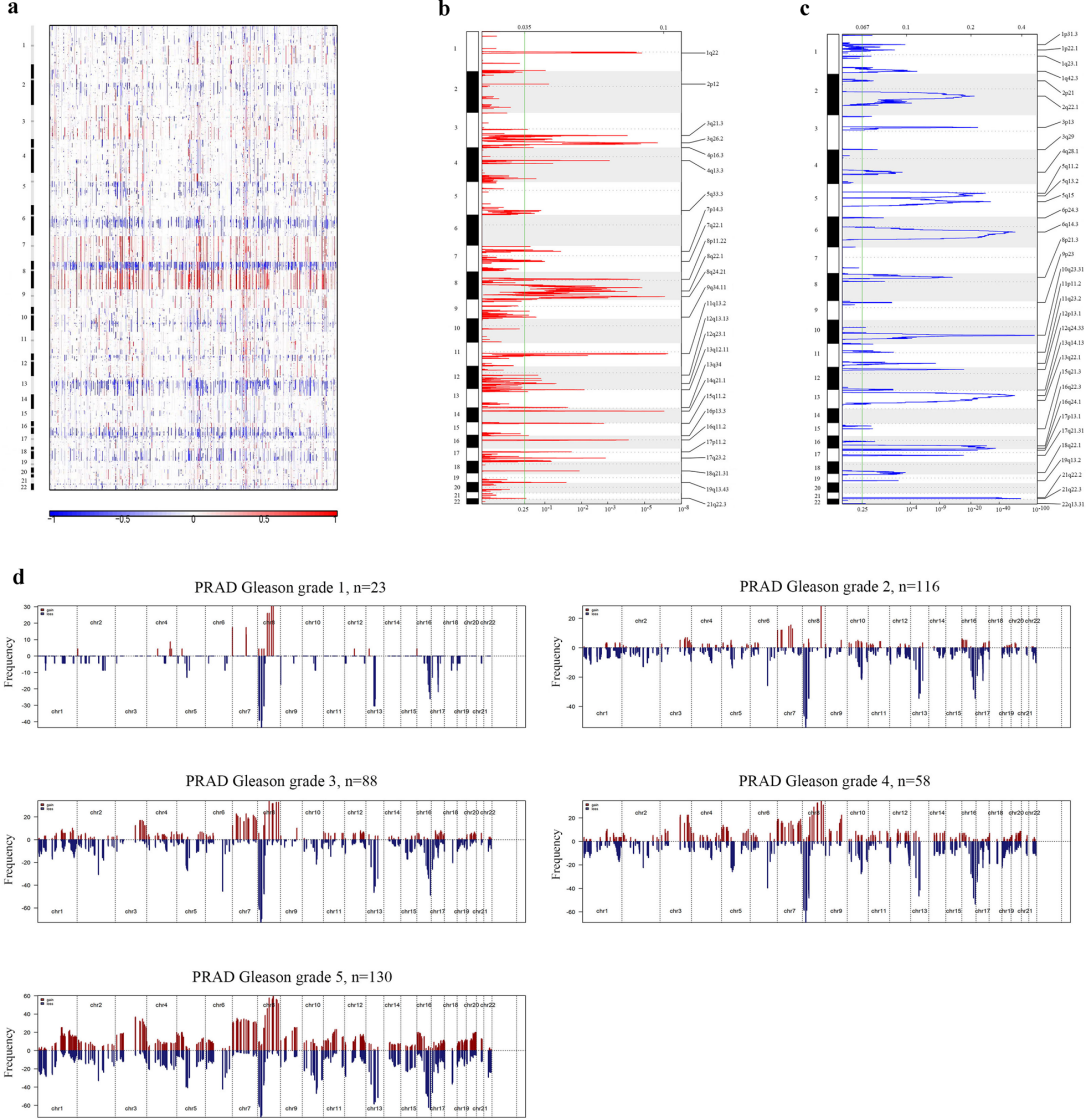
The stem gene CNV is related to cell stemness and malignancy of prostate cancer. **a–c** Changes of stemness gene CNV in PCa samples. Red and blue represented the two types of CNV, amplification and deletion, respectively. **d** The stem gene CNV increased with the increase of clinical Gleason grade. Red and blue represented the two types of CNV, amplification and deletion, respectively

The segments containing all MEmagenta module genes were obtained from TCGA and then analyzed by using GISTIC (Genome Identification of Significant Targets in Cancer). Analytical results showed that most of MEmagenta module genes had higher variations in prostate cancer than those in normal prostate tissue, indicating that the stemness of PCa cells was influenced by the CNVs (Copy Number Variations) of these genes (Fig. 2a and Additional file 4: Fig. S2a). In the two kinds of gene CNVs, amplification and deletion (Additional file 5: Table S3 and Additional file 6: Table S4), the changes of deletion CNVs were more than those of amplification CNVs in the MEmagenta module genes of PCa samples; and both amplification and deletion CNVs in PCa samples were much higher than those in prostate normal samples (Fig. 2b, c, and Additional file 4: Fig. S2b, c). By jointly analyzing GISTIC results with Gleason grade of PCa, it’s found that CNVs of MEmagenta module genes also increased with the Gleason grade (Fig. 2d), suggesting that CNVs of MEmagenta module genes were highly related to the malignancy of PCa.

In details of CNVs of some genes in the MEmagenta module, it was found that the CNVs (amplification) and expression of SKA3 and RUVBL1, which promoted tumor metastasis and played a role in the development of stem cells [37–40], were all increased with the Gleason grade (Additional file 7: Fig. S3a. b); and the CNVs (deletion) and expression of MCM6 and CENPH, which enhanced cancer cell proliferation, stemness, and metastasis and promoted cancer development [41–45], were also increased with the increase of Gleason grade (Additional file 7: Fig. S3c, d).

### Transcription factors of stemness-related genes enhanced the stemness of PCa cells

**Fig. 3:**
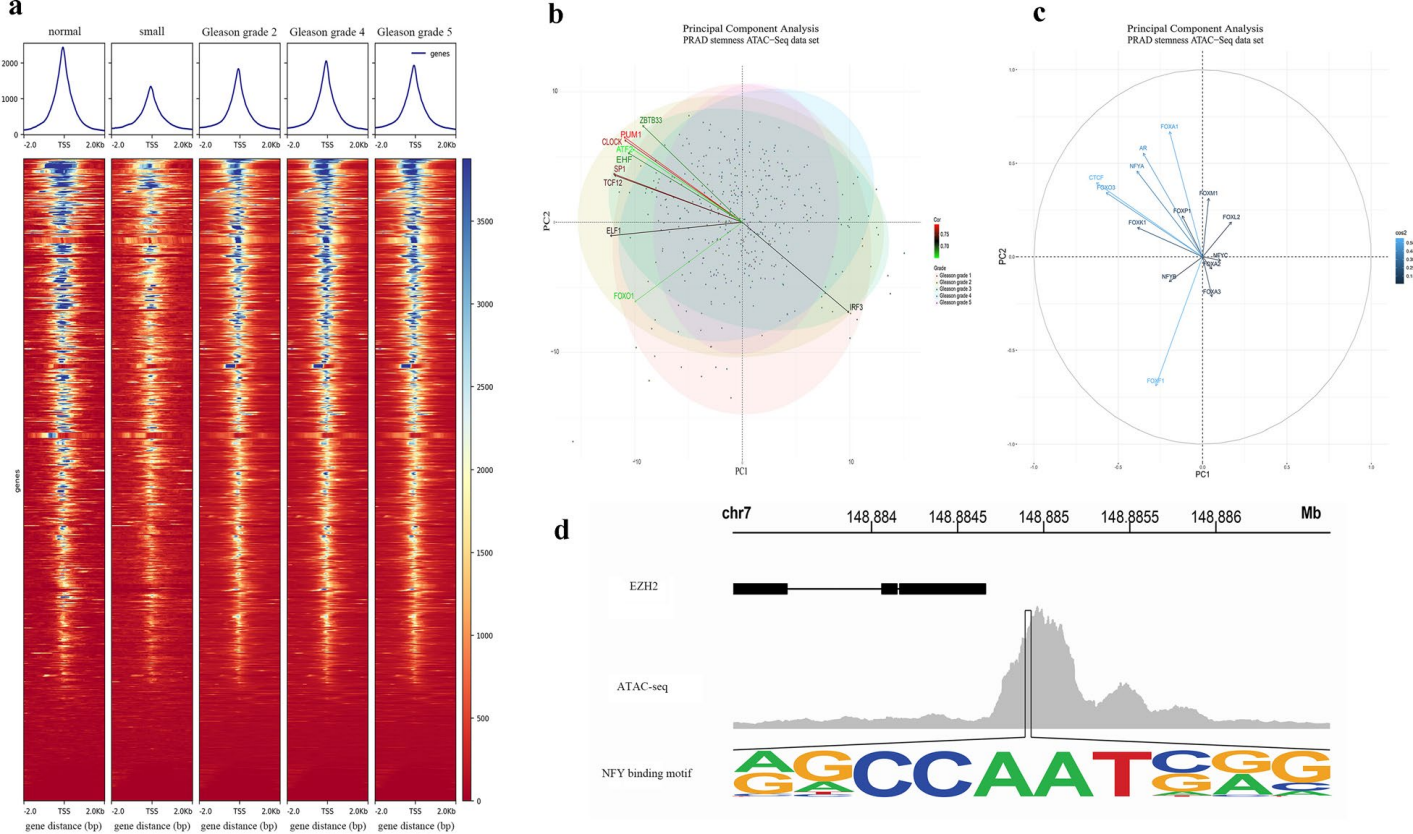
Transcriptional regulation of stemness gene in prostate cancer. **a** The transcription factor binding intensity on stemness genes in normal prostate tissue samples, PCa tissue samples with different Gleason grades and prostate small cell cancer tissue samples. **b** The main transcriptional regulators were obtained by using PCA analysis. Red and green represent the strength and weakness of transcriptional regulatory factors, respectively. **c** The importance of known transcriptional regulators of stemness genes in prostate cancer was obtained by using PCA analysis, The shades of blue represent the correlation between transcription factors and stemness. **d** Motif map of transcription factors binding to EZH2

We downloaded the ATAC-seq data of the MEmagenta module genes in PCa samples from TCGA and analyzed the sequence data of the 2 kb range of the transcription start site (TSS). From the results, although both normal and tumor prostate samples had the open chromatin signal, the open chromatin signal of all PCa samples (including the different Gleason grades of PCa and small cell PCa) was weaker than that of the normal prostate samples (Fig. 3a); and the open chromatin signal in small cell PCa was the weakest one (Fig. 3a). These results indicated that the open chromatin signal of the MEmagenta module genes in PCa might differ from those in normal prostate tissues.

According to the ATAC-seq data analysis results, it was found that most transcription factors of stemness genes in prostate samples were the same (Additional file 8: Fig. S4b). After filtering the 238 common transcription factors according to the genome information and transcriptome data, the expression profile of 163 transcription factors was finally obtained. According to the expression of the 163 transcription factors, 3 clusters were obtained by clustering the transcriptome data of the 163 transcription factors (Additional file 8: Fig. S4a). The first cluster had 86 transcription factors (such as FOXA1, HOXB13, ERG1, et al.), the second cluster had 44 transcription factors (such as SOX15, FOXM1, SOX2, et al.), and the third cluster had 33 transcription factors (such as NANOG, SOX3, FOXA2, et al.) (Additional file 8: Fig. S4a). In the top ten results of principal component analysis (PCA) of the 163 transcription factors, stemness-related PUM1 [46, 47], CLOCK [48], SP1, and TCF12 played a major positive regulatory role in PCa cell stemness (Fig. 3b and Additional file 8: Fig. S4c). In comparison, IRF3 [49] negatively correlated with other 9 transcription factors and played a negative regulatory role in PCa cell stemness (Fig. 3b). As we know, IRF3 (interferon regulatory factor 3) signaling played an essential role in TLR3-mediated apoptosis in LNCaP cells through the activation of the intrinsic and extrinsic apoptotic pathways [49], suggesting that the immune system might play a role in suppressing the stemness of PCa cells. Furthermore, our analysis results showed that AR, FOXA1, NFYA, CTCF, and FOXO3 might enhance the stemness of PCa cells, while FOXF1 might be negatively correlated with these transcription factors (Fig. 3c).

In normal prostate samples, we found that the major transcription regulators of stemness genes were HOXB4, NFYB, and TFE3 (Additional file 9: Fig. S5a, c), which differed from those in prostate cancer. From the PCA analysis results, we found that the roles of FOXA1, NFYA, and FOXP1 in regulating stemness genes in normal prostate samples were changed by comparing those in prostate cancer samples (Additional file 9: Fig. S5b and Fig. 3c). These results indicated that the same transcription factors might play different roles in regulating the cell stemness in prostate normal and cancer samples.

Our results of transcriptional regulator analysis showed that the upstream of the EZH2, the most relevant gene to the stemness of PCa cells, could be significantly bound by NFY (Fig. 3d). As a transcription factor, NFY regulates the self-renewal of hematopoietic stem cells [51] and promotes the self-renewal and expansion of prostate cancer cells and their stemness [50]. Hence EZH2 might play a stemness role in prostate cancer.

### Immunological microenvironment negatively related to the stemness of PCa cells

**Fig. 4:**
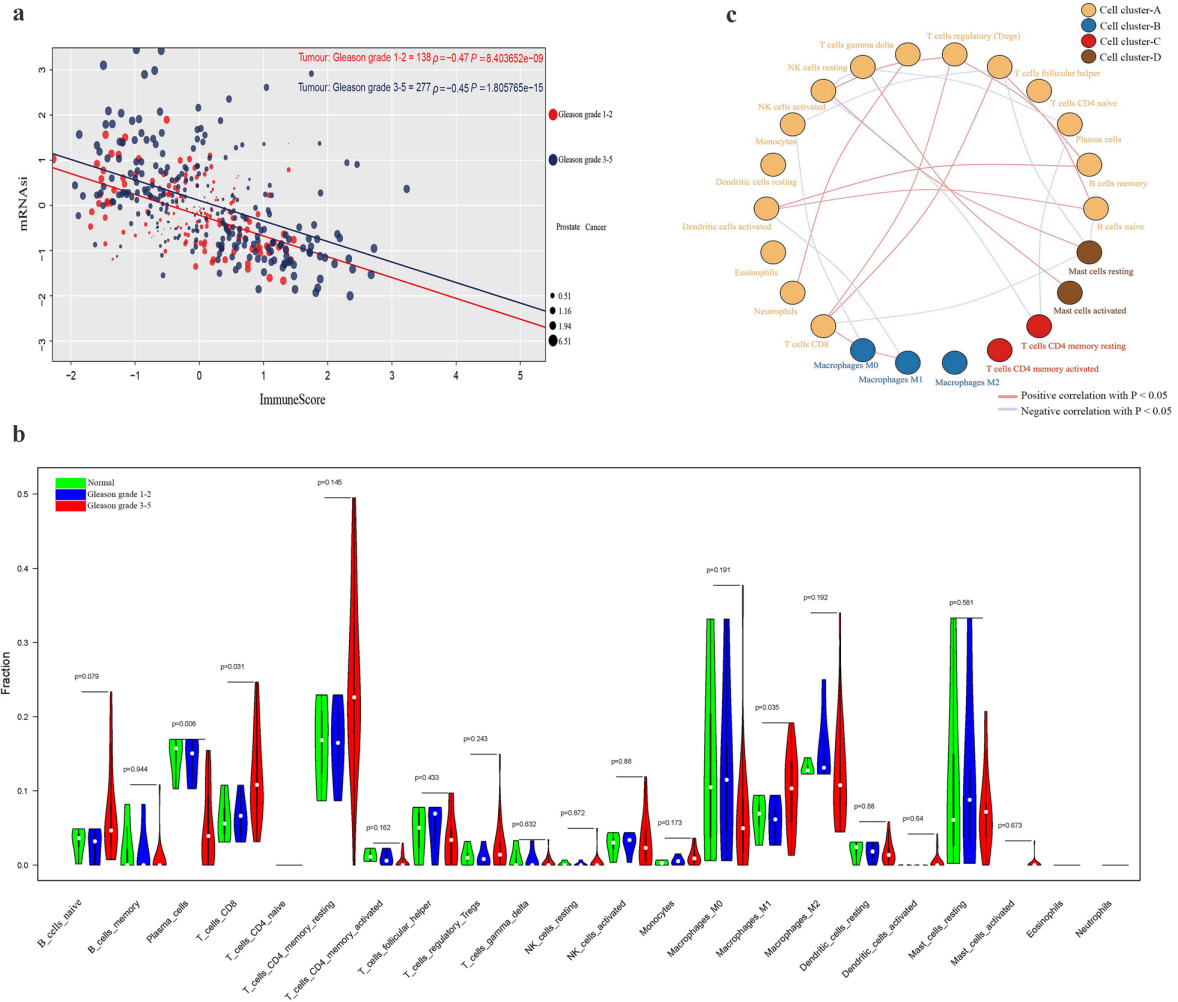
Stemness and immune cells in prostate cancer. **a** Stemness score was negatively correlated with immune infiltration score in prostate cancers with different Gleason grades. **b** The fraction of 22 types of immune cells in the microenvironment of prostate cancer with different Gleason grades. **c** Correlation network of 22 types of immune cells

By scoring the stemness and immunity of the immune microenvironment of PCa with different Gleason grades in clinical samples, there was a negative correlation between the PCa cell stemness and the immunity of the microenvironment of PCa in all clinical samples. The correlation coefficient between stemness and immunity of the immune microenvironment in PCa with Gleason grade 1–2 was almost the same as that in PCa with Gleason grade 3–5 (Fig. 4a). By analyzing and scoring the stromal and immune cells in the PCa microenvironment, we found that the scores of stromal and immune cells in the PCa microenvironment were all inversely related to the stemness of PCa cells (Additional file 10: Fig. S6a). Although the immune score generally increased with the increase of Gleason grade, the stemness of PCa samples with low-immunity was significantly higher than that of PCa samples with high-immunity in the same Gleason grade PCa samples (Additional file 10: Fig. S6b). After clustering the MEmangenta module genes based on cell stemness and immune scores of PCa, we found that PCa cell stemness was negatively related to the immunity of the PCa microenvironment in the clinical samples with high expression of the MEmagenta module genes (Additional file 10: Fig. S6d).

By analyzing and scoring the transcriptome data from TCGA for the immune infiltration of PCa clinical samples, the number of 22 types of immune cells in prostate cancer with different Gleason grades was obtained (Fig. 4b). From the results, it’s found that most types of immune cells in the microenvironment of PCa were increased with the Gleason grade increase; and both CD8 + T cells [8] and macrophage M1 [9] were significantly increased in all types of immune cells, while the plasma cells [51] were reduced with the Gleason grade increase (Fig. 4b), indicating that plasma cells (B cells) in the microenvironment of PCa played a vital role in anti-PCa immunity.

Based on the correlation between different types of immune cells, immune cells were divided into 4 clusters by using clustering method. There were 15 immune cells in cluster-A, 3 immune cells in cluster-B, 2 immune cells in cluster-C and 2 immune cells in cluster-D; and correlation between different immune cells in cluster-A was more than that in other clusters, suggesting that cluster-A was more complicated in this immune cell network (Fig. 4c and Additional file 11: Table S5). For example, there was a negative correlation between activated NK cells and resting NK cells, which is most significant among different types of immune cells (Additional file 10: Fig. S6c), and the number of activated NK cells increased with the Gleason grade increase in PCa (Fig. 4b), indicating that activated NK cells might play a significant inhibitory function on prostate cancer stemness. The most significant positive correlation was between the activated dendritic cells and the memory B cells, and the number of both activated dendritic cells and memory B cells was all increased with the Gleason grade increase (Fig. 4c), which indicated that these two types of immune cells might play essential roles in inhibiting the stemness of PCa cells.

### The protein interaction network of stemness-related genes and its relationship with prostate cancer immune cells

**Fig. 5:**
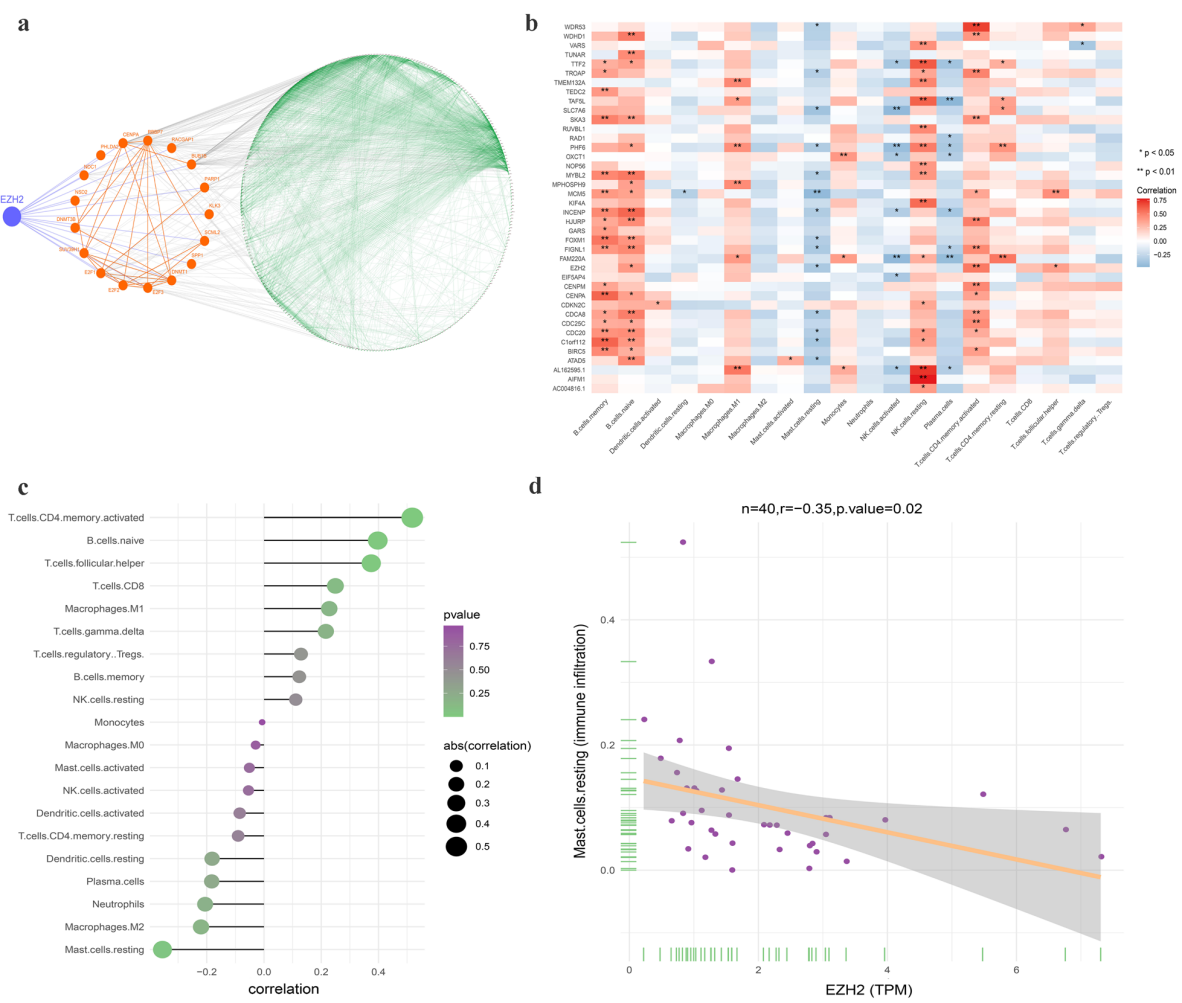
The protein interaction network of stemness gene and the relationship between stemness gene and immune infiltration. **a** The connection between stemness genes and EZH2 in MEmagenta module. According to whether the gene interacted with EZH2 directly or not, it could be classified into two groups: genes in small circle and genes in large circle. The blue lines represented the genes that directly interacted with EZH2, the orange red lines represented the interaction between genes in the small circle, the green lines represented the interaction between genes in the large circle and the gray lines represented the interaction of genes between the small circle and the large circle. **b** The correlation between stemness gene and immune cells. Red represented positive correlation; blue represented negative correlation. **c, d** The correlation between EZH2 and immune cells and the correlation between EZH2 and resting mast cell, respectively

By screening and analyzing human protein interaction data containing MEmagenta module genes from the STRING database, we not only constructed the important protein–protein interaction network in the proteins of MEmagenta module genes, but also found that EZH2 interacted directly with 17 proteins. In the EZH2-related 17 protein–protein interactions, we also found that EZH2 could regulate the entire protein interaction network of MEmagenta module stemness genes by mainly interacting with CENPA, BUB1B, and PARP1 (Fig. 5a) [52–55]. Furthermore, function enrichment analysis of the MEmagenta module stemness genes revealed that most functions of these genes were concentrated in cell mitosis, and the most significant functional pathway was related to cell cycle (Additional file 12: Fig. S7a, b), suggesting that these stemness genes might involve in the regulation of PCa stem cell mitosis.

From the analysis results of the relationship between the expression of the MEmagenta module stemness genes and immune infiltration of PCa, we found that different immune cells had different effects on the expression of stemness genes in PCa. Different types of immune cells could affect the same stemness gene expression, and one stemness gene expression could also reversely affect different types of immune cells (Additional file 13: Table S6). In the correlations between the expression of stemness genes and immune cells, we found that expression of most stemness genes was positively correlated to memory B cells and naive B cells and negatively correlated to plasma cells (Fig. 5b). With the increase of PCa Gleason grade, the number of B cells increased and the number of plasma cells decreased. (Fig. 4b). These results indicated that B cells might play the opposite effects on PCa cell stemness in different conditions (it's consistent with reference [56]). Besides, the number of activated NK cells and memory CD4 + T cells were all increased with the PCa Gleason grade increase; and the expression of the MEmagenta module stemness genes was positively correlated to resting NK cells and memory CD4 + T cells, while negatively correlated to activated NK cells (Figs. 4b and 5b).

Expression of EZH2, the most relevant gene to PCa cell stemness, was most positively correlated to the activated memory CD4 + T cells and negatively correlated to the resting Mast cells (Fig. 5c, d). Most types of immune cells positively correlated to the expression of the EZH2 were T cells and B cells (Fig. 5c), suggesting that T cells and B cells were the critical immune cells in regulating PCa cell stemness by controlling the expression of the EZH2. In addition, with the increased PCa Gleason grade, the number of resting mast cells decreased and the number of activated mast cells increased (Fig. 4b). The resting mast cells were the most negatively correlated to the expression of the EZH2 (Fig. 5c, d). These results indicated that the immunity and immune cells of the microenvironment of PCa played an essential role in the tumorigenesis and development of prostate cancer.

## Discussion

It had been identified that cancer stem cells were closely related to cancer cell migration and played critical roles in the metastasis of PCa in clinic [57]. The research on the stemness of PCa cells and their influencing factors was inevitably becoming a key direction of tumor research. In this study, we found the gene module (MEmagenta module) most related to stemness of prostate cancer cells by analyzing multi-omics of PCa clinical samples and cell lines, and also found that the CNVs and transcriptional regulators of the MEmagenta module genes were the critical influencing factors related to the stemness of PCa cells by conjoint analysis of transcriptome data, gene CNV data and ATAC-seq data of prostate cancer. In the analysis results of the immune microenvironment of prostate cancer, it's found that the immune microenvironment of PCa negatively correlated with the stemness of PCa cells, which played an important role in the stemness of PCa cells. The immune cells in the immune microenvironment of PCa had different correlations with the expression of stemness genes of the MEmagenta module, suggesting that the immune microenvironment of PCa and its immune cells were also the essential influencing factors related to the stemness of PCa cells.

It's reported that tumor cell stemness could affect the immune response of the tumor immune microenvironment and then result in tumor heterogeneity [32]. Although there have been many studies on the relationship between tumor cell stemness and tumor development in other tumors, few studies on the relationship between tumor cell stemness and PCa development, especially on the factors affecting and regulating the cell stemness of prostate cancer. In this study, by fully using a variety of bioinformatics analysis software and methods to analyze multiple omics data, we not only obtained two factors related to PCa cell stemness, including the CNVs of stemness-related genes and the immunity of tumor microenvironment, but also got the gene set most relevant to stemness of PCa cells. Many critical genes included in this gene set and their transcription regulators were important in regulating and affecting PCa metastasis via influencing PCa cell stemness.

EZH2 not only promotes the formation of cancer stem cells, but also expands the aggressive cancer cell population and leads to cancer progression [58]. EZH2 can also co-regulate prostate cancer stem cell properties with BRCA1 [33]. RUBVL1 is a gene relevant to the stemness of prostate cancer cells, and its copy number increases with the tumor progresses. It is reported that RUVBL1 is essential for the survival of hematopoietic stem cells [40], and its gene copy number is increased in head and neck squamous cancers [59]. MYBL2 can help DNA double-strand repair in hematopoietic stem cells [60] and the emergence of CNV leads to the occurrence and development of cancer [61, 62]. AURKB could determine the identity of embryonic stem cells [63], and the CNV (deletion) also contributes to the formation of aggressive tumors [64]. PUM1 regulates the expression of hematopoietic stem cells [65] and promotes the migration of cancer cells [47]. CLOCK regulates the biological clock of cancer stem cells and promotes the self-renewal of cancer cells [66]. HMGCS1 can promote cancer development [67] and affect the function of NK cells [68]. SUV39H1 attenuates the apoptosis of cancer cells [69] and enhances the immune escape of tumor cells [70]. In addition to the reported genes related to cancer cell stemness, our results also showed many genes that had not been reported to be related to cancer cell stemness, especially to PCa cell stemness, such as TEDC2, TMEM132A, and VARS, etc.

Up to now, there are few studies on the relationship between cell stemness regulatory factors and tumor malignancy in PCa. From our multi-omics analysis results, the CNVs of cancer cell stemness-related genes were closely related to the malignant degree of prostate cancer (Gleason grade). The CNVs of genes positively related to PCa cell stemness were also positively correlated with the degree of malignant prostate cancer (Gleason grade); conversely, the CNVs of genes negatively associated with PCa cell stemness were also negatively correlated with Gleason grades of prostate cancers. The correlations between transcriptional regulators of PCa cell stemness genes and prostate tumor malignancy were similar to the correlation between CNVs of PCa cell stemness genes and prostate tumor malignancy.

Although we found that the CNVs and transcriptional regulations of PCa cell stemness genes and the immune infiltration of prostate cancer were all important factors in influencing the stemness of PCa cells and further regulating the development of PCa and we also analyzed and obtained the key gene set and genes in regulating the stemness of PCa cells, the experimental evidence and the detailed mechanism of the correlation and regulation among CNVs of PCa cell stemness genes, transcription regulators of PCa cell stemness genes and immunity of PCa microenvironment were still unclear and needed to be further investigated in the future.

## Conclusions

Prostate cancer samples with high Gleason grade had high CNVs of stemness genes and high stemness score. Low immunity of immune microenvironment was beneficial to the stemness of prostate cancer cells. Stemness of PCa cells was positively correlated with the malignancy of PCa. The transcription factors of most of important stemness genes were important in promoting the stemness of PCa cells via enhancing the expression of stemness genes in prostate cancer. However, its detailed molecular mechanism still needed to be future identified with in vivo and in vitro experiments.

## Supplementary Information

**Additional file 1: Figure S1. Screen and verification of stemness gene modules by WGCNA analysis. a **WGCNA analysis results. Branches with different colors corresponding to different modules. **b** Analysis of the correlation among genes, modules and stemness. **c** The stemness gene obtained from jointly analyzing WGCNA analysis results and the most known stemness gene markers. **d** and **e** The expression of stemness genes in prostate cancer tissues and cell lines, respectively.

**Additional file 2.** Table S1. The relationship between genes and color modules in WGCNA analysis results.

**Additional file 3.** Table S2. Results of differential expression of stemness genes in MEmagenta module.

**Additional file 4: Figure S2. CNVs of MEmagenta module stemness genes in normal prostate tissue samples. a** Overview of CNV in normal prostate tissue samples. **b **and **c** The CNVs with amplification or with deletion, respectively.

**Additional file 5.** Table S3. The result of copy number amplification in the genome location of the stemness genes in the MEmagenta module.

**Additional file 6.** Table S4. The result of copy number deletion in the genome location of the stemness genes in the MEmagenta module.

**Additional file 7: Figure S3 CNVs and expression of stemness genes all increased with the increase of Gleason grades. a** and **b** The expression and CNVs (amplification) of SKA3 and RUVBL1 increased with increase of Gleason grades. **c **and **d** The expression and CNVs (deletion) of MCM6 and CENPH increased with increase of Gleason grades.

**Additional file 8: Figure S4 Transcriptional regulators of stemness genes in prostate cancer. a** Expression heatmap of transcriptional regulators of stemness genes in PCa samples with different Gleason grades. **b** Venn diagram of transcriptional regulators of stemness genes in in PCa samples with different Gleason grades. **c** The importance of transcriptional regulators of stemness genes in prostate cancer was obtained by using PCA analysis.

**Additional file 9: Figure S5 Analysis of transcriptional regulators of stemness genes in normal samples. a** The main transcriptional regulators were obtained by using PCA analysis. **b** Based on PCA analysis, the importance of known transcriptional regulators of stemness genes in normal samples was obtained. **c** Based on PCA analysis, the importance of transcriptional regulators of stemness genes in normal samples was obtained.

**Additional file 10: Figure S6 Prostate tumor immune microenvironment is negatively correlated with stemness. a** Stromal cell score and immunity score of the PCa microenvironment were negatively correlated with PCa cell stemness score. **b** The immune score increased with the Gleason grade increase, and the low immunity score had a higher stemness score in PCa with different Gleason grades. **c** The correlations between different types of immune cells in 22 types of immune cell. **d** The expression heatmap of stemness genes in PCa samples with high/low immune score, different stemness score, and different Gleason grade.

**Additional file 11.** Table S5. Results of correlation between different immune cells.

**Additional file 12: Figure S7 GO and KEGG enrichment analysis of stemness gene in MEmagenta module. a** The results of GO enrichment analysis. **b** The results of KEGG enrichment analysis.

**Additional file 13.** Table S6. Correlation results between stemness genes and different immune cells in the MEmagenta module.

## Data Availability

All data generated or analyzed during this study are available.

## Abbreviations

pCSC: Prostate cancer stem cells
PCa: Prostate cancer
WGCNA: Weighted gene co-expression network analysis
STRING: Functional protein association networks
CNVs: Copy number variants
CSCs: Cancer stem cells
PPI: Protein–protein interaction
SCPC: Small cell prostate cancer
PRAD: Prostate adenocarcinoma
