# Supplementary figures and images for: High copy number variations, particular transcription factors, and low immunity contribute to the stemness of prostate cancer cells
Source: J Transl Med. 2021 May 13;19:206. doi: 10.1186/s12967-021-02870-x (PMC8117623; doi:10.1186/s12967-021-02870-x)

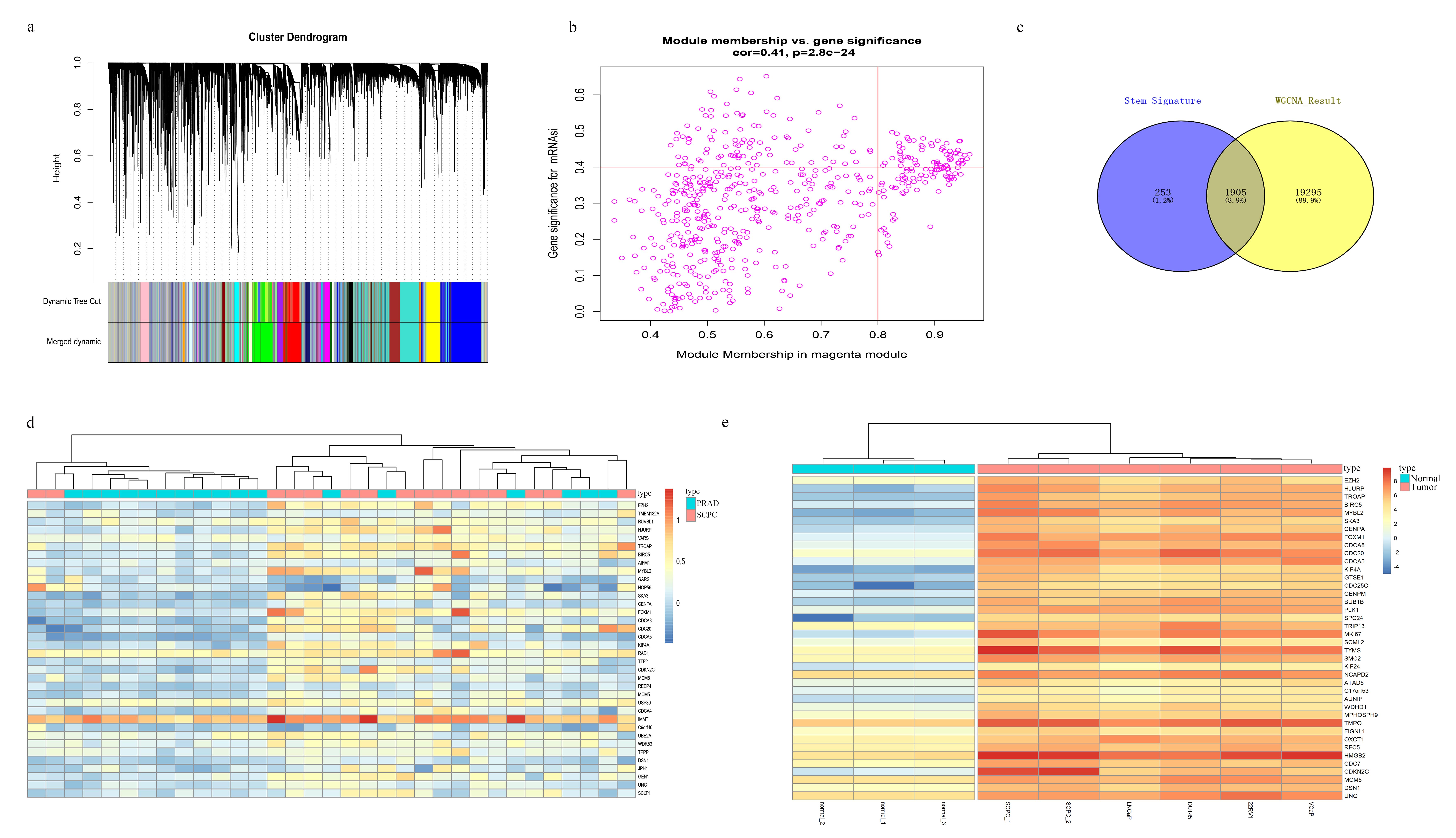

Supplement: Supplementary file 1 — Additional file 1: Figure S1. Screen and verification of stemness gene modules by WGCNA analysis. a WGCNA analysis results. Branches with different colors corresponding to different modules. b Analysis of the correlation among genes, modules and stemness. c The stemness gene obtained from jointly analyzing WGCNA analysis results and the most known stemness gene markers. d and e The expression of stemness genes in prostate cancer tissues and cell lines, respectively. [file 12967_2021_2870_MOESM1_ESM.jpg]

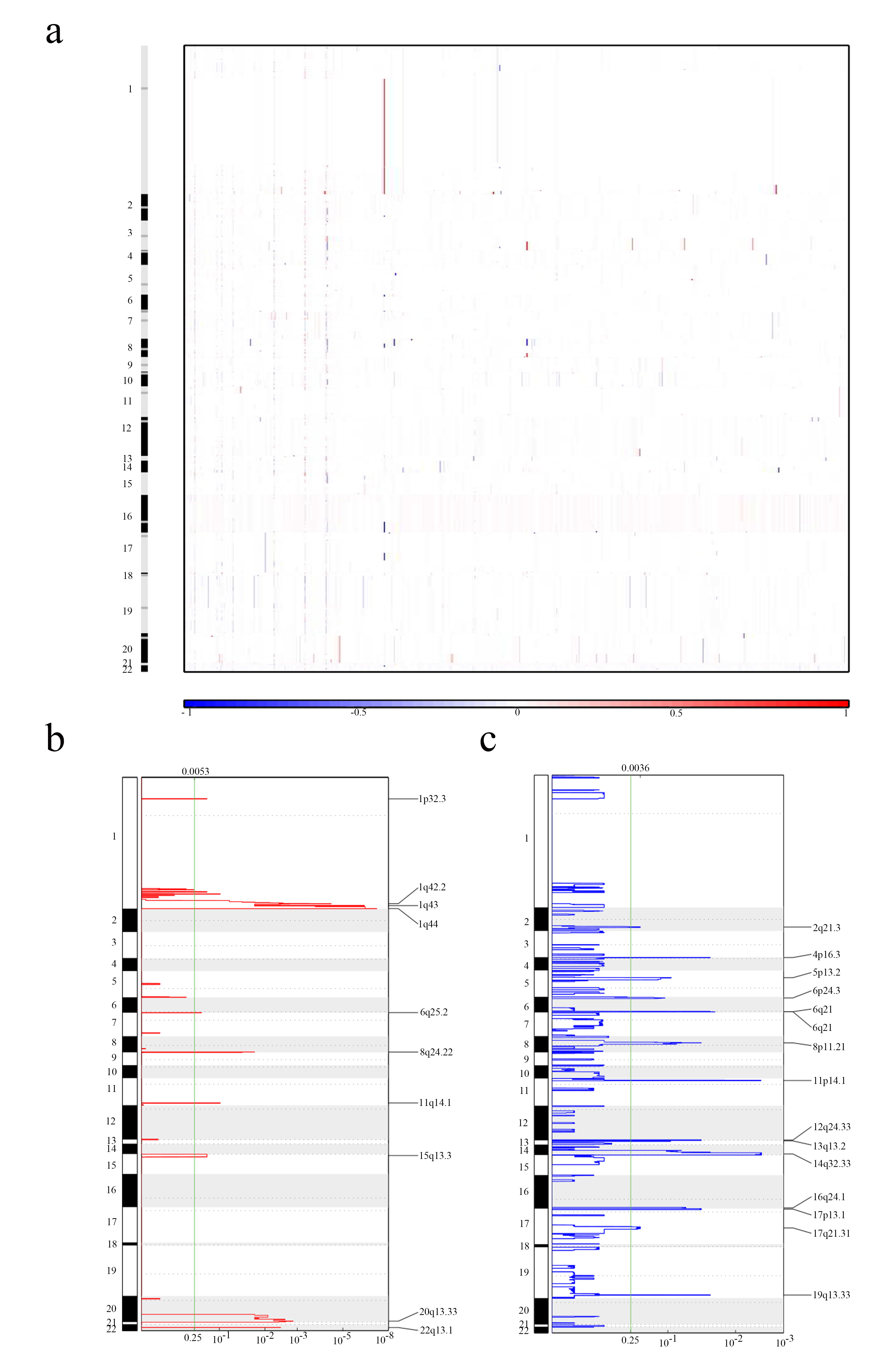

Supplement: Supplementary file 4 — Additional file 4: Figure S2. CNVs of MEmagenta module stemness genes in normal prostate tissue samples. a Overview of CNV in normal prostate tissue samples. b and c The CNVs with amplification or with deletion, respectively. [file 12967_2021_2870_MOESM4_ESM.jpg]

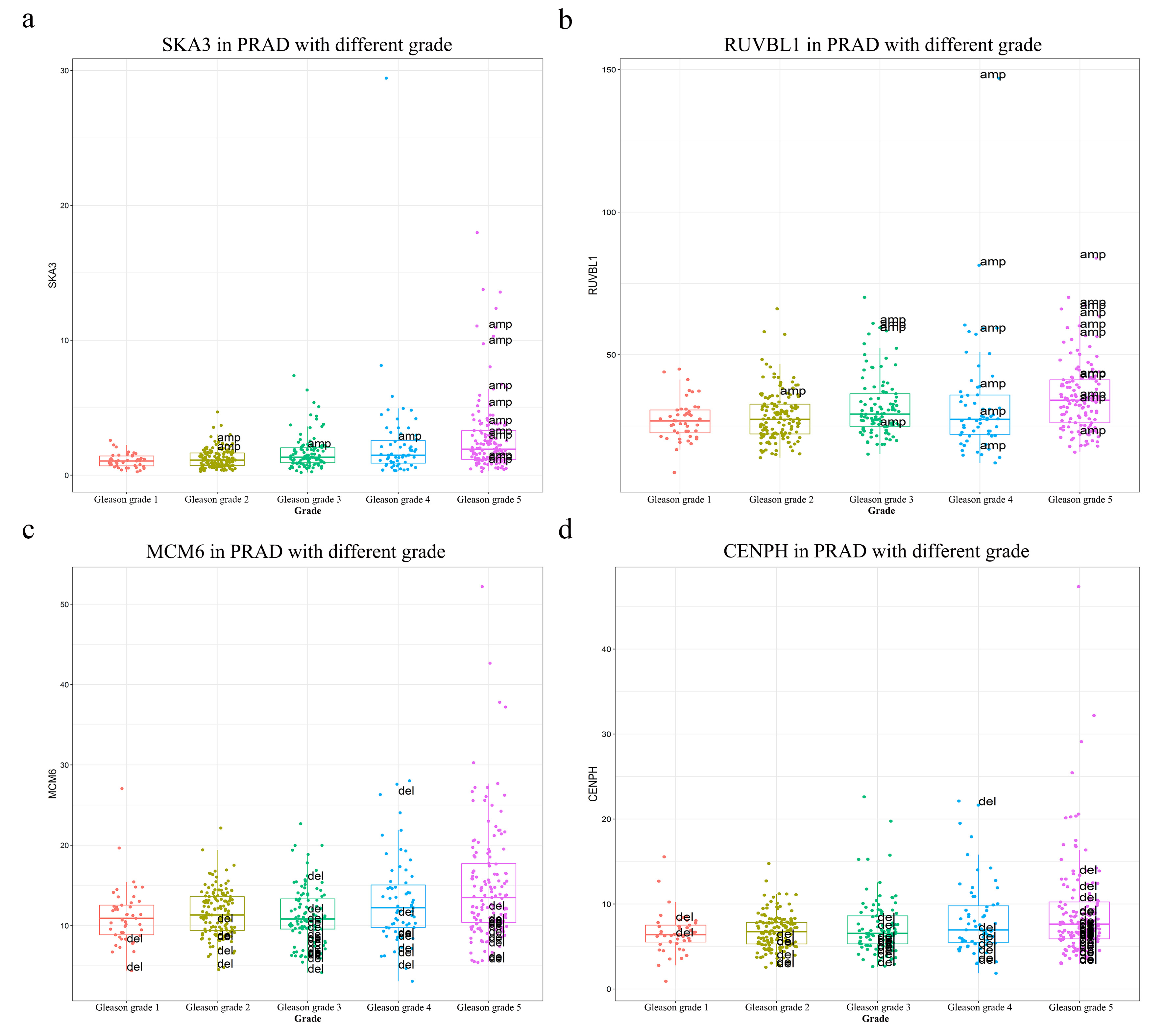

Supplement: Supplementary file 7 — Additional file 7: Figure S3 CNVs and expression of stemness genes all increased with the increase of Gleason grades. a and b The expression and CNVs (amplification) of SKA3 and RUVBL1 increased with increase of Gleason grades. c and d The expression and CNVs (deletion) of MCM6 and CENPH increased with increase of Gleason grades. [file 12967_2021_2870_MOESM7_ESM.jpg]

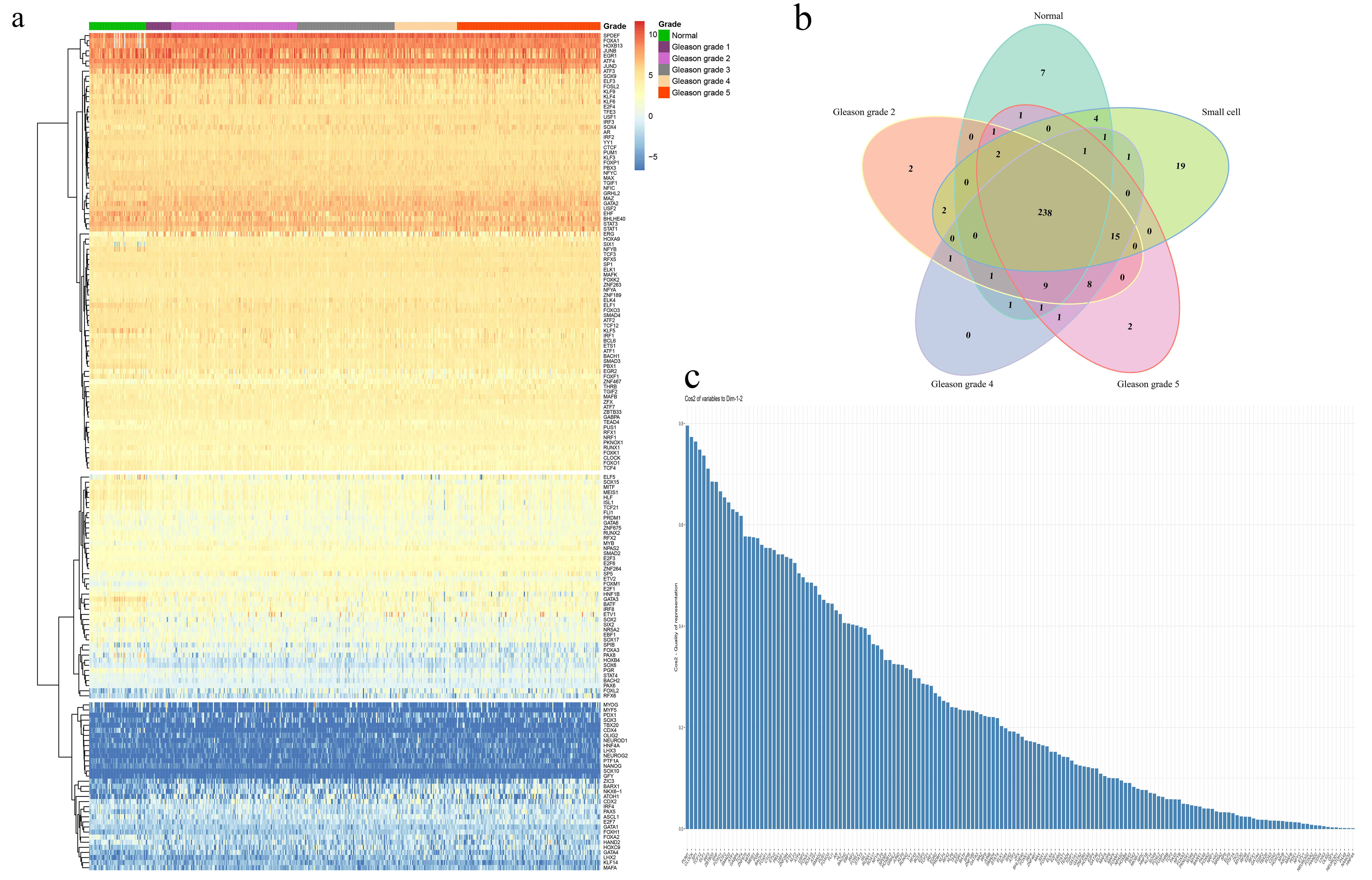

Supplement: Supplementary file 8 — Additional file 8: Figure S4 Transcriptional regulators of stemness genes in prostate cancer. a Expression heatmap of transcriptional regulators of stemness genes in PCa samples with different Gleason grades. b Venn diagram of transcriptional regulators of stemness genes in in PCa samples with different Gleason grades. c The importance of transcriptional regulators of stemness genes in prostate cancer was obtained by using PCA analysis. [file 12967_2021_2870_MOESM8_ESM.jpg]

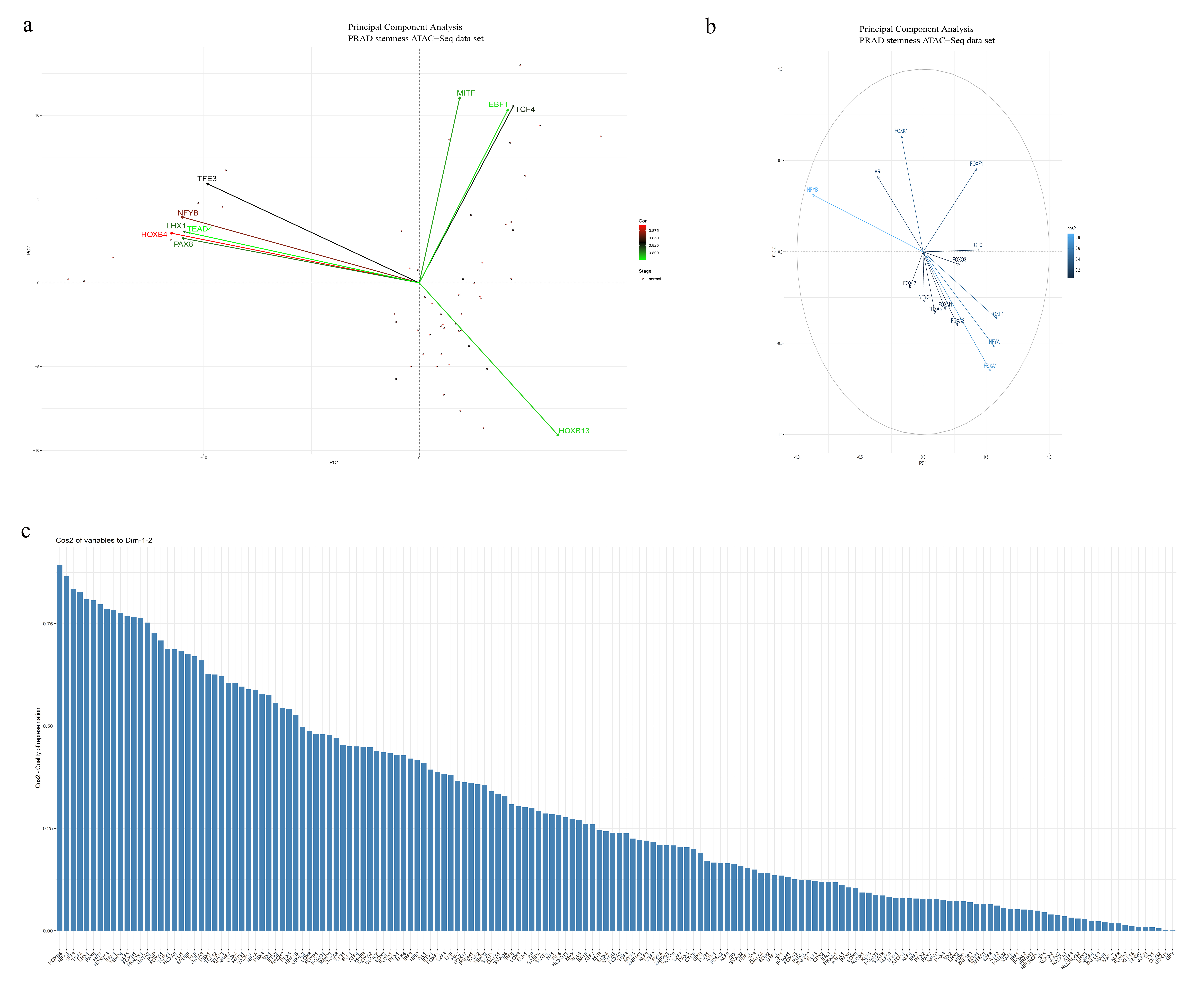

Supplement: Supplementary file 9 — Additional file 9: Figure S5 Analysis of transcriptional regulators of stemness genes in normal samples. a The main transcriptional regulators were obtained by using PCA analysis. b Based on PCA analysis, the importance of known transcriptional regulators of stemness genes in normal samples was obtained. c Based on PCA analysis, the importance of transcriptional regulators of stemness genes in normal samples was obtained. [file 12967_2021_2870_MOESM9_ESM.jpg]

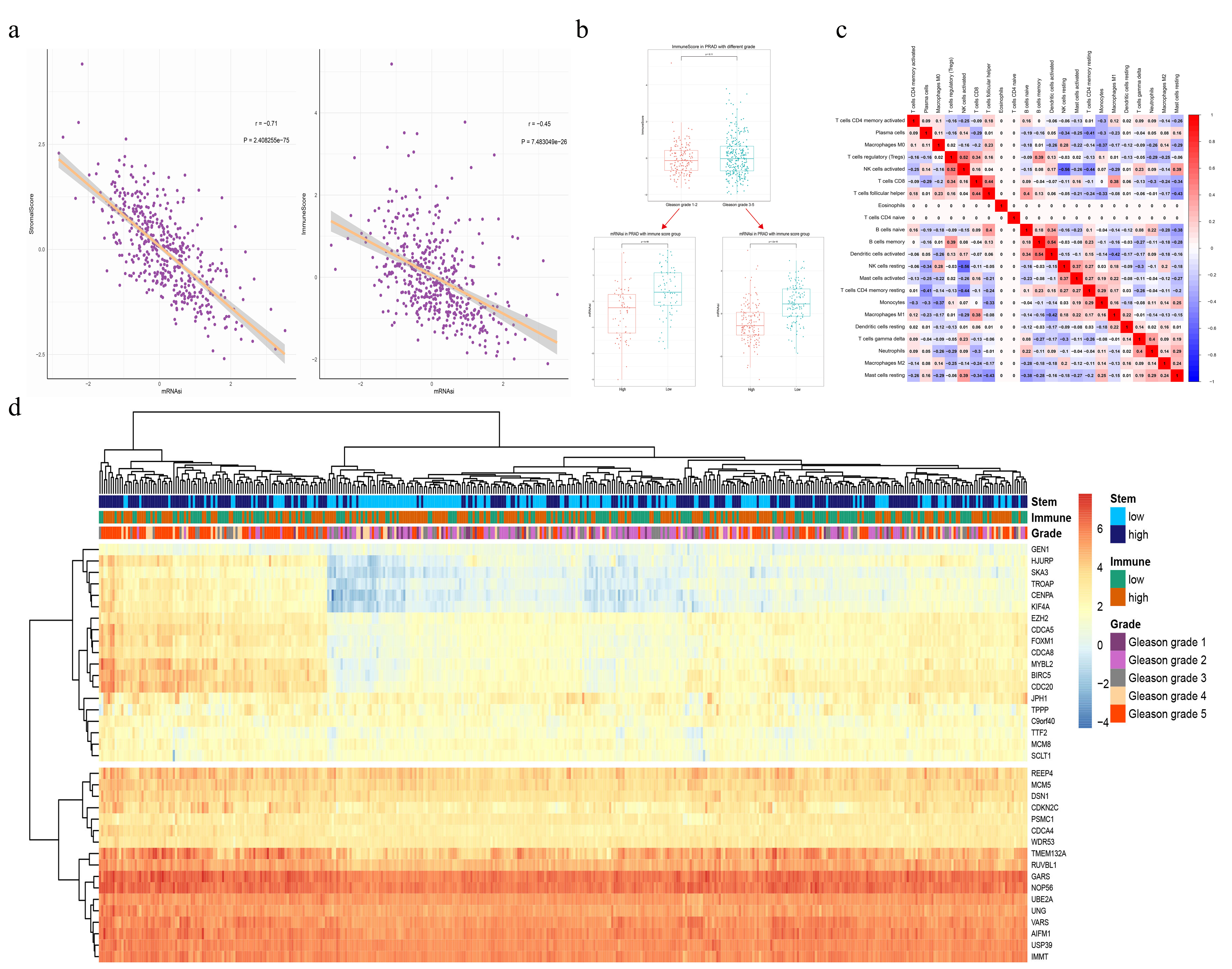

Supplement: Supplementary file 10 — Additional file 10: Figure S6 Prostate tumor immune microenvironment is negatively correlated with stemness. a Stromal cell score and immunity score of the PCa microenvironment were negatively correlated with PCa cell stemness score. b The immune score increased with the Gleason grade increase, and the low immunity score had a higher stemness score in PCa with different Gleason grades. c The correlations between different types of immune cells in 22 types of immune cell. d The expression heatmap of stemness genes in PCa samples with high/low immune score, different stemness score, and different Gleason grade. [file 12967_2021_2870_MOESM10_ESM.jpg]

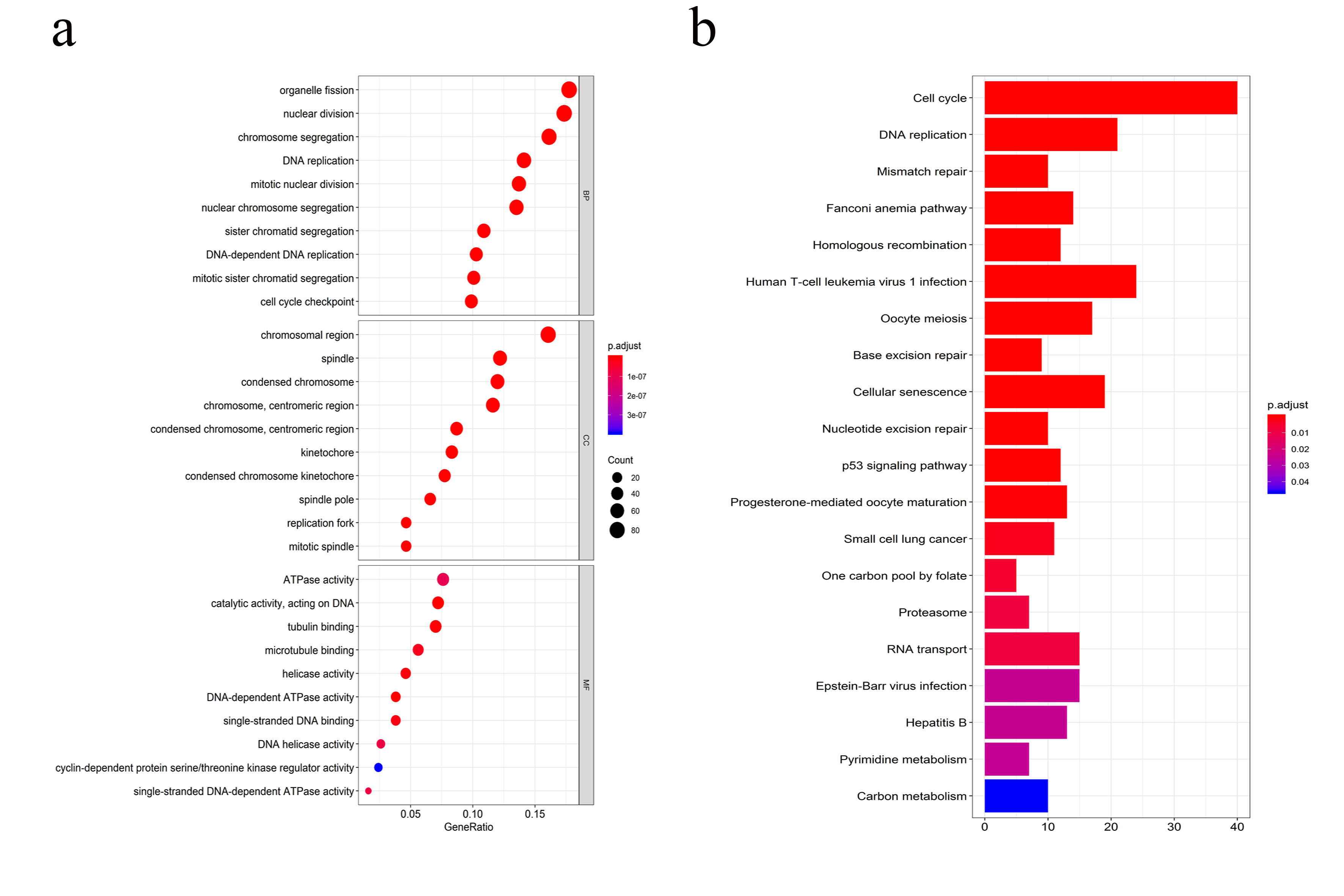

Supplement: Supplementary file 12 — Additional file 12: Figure S7 GO and KEGG enrichment analysis of stemness gene in MEmagenta module. a The results of GO enrichment analysis. b The results of KEGG enrichment analysis. [file 12967_2021_2870_MOESM12_ESM.jpg]
